# Correction: Population genetic diversity and structure of the endangered species *Tetracentron sinense* Oliver (Tetracentraceae) with SNPs based on RAD sequencing

**DOI:** 10.1371/journal.pone.0331705

**Published:** 2025-09-04

**Authors:** The PLOS One Staff

There are errors in the author affiliations. The correct affiliations are as follows:

Zhong–Qiong Tian^1,2^, Chao–Yang Jiang^1^, Yu–Min Shu^1^, Huan Zhang3, Qiong La^1^, Xiao-Hong Gan^1,3^

**1** Key laboratory of southwest China wildlife resources conservation (Ministry of Education), China West Normal University, Nanchong, China, **2** Key Laboratory of Biodiversity and Environment on the QinghaiTibetan Plateau, Ministry of Education, Lhasa, China, **3** Institute of plant adaptation and utilization in southwest mountain, China West Normal University, Nanchong, China.

The publisher apologizes for the error.
