# Supplementary figures and images for: Population genetic diversity and structure of the endangered species Tetracentron sinense Oliver (Tetracentraceae) with SNPs based on RAD sequencing
Source: PLoS One. 2025 May 20;20(5):e0324161. doi: 10.1371/journal.pone.0324161 (PMC12091802; doi:10.1371/journal.pone.0324161)

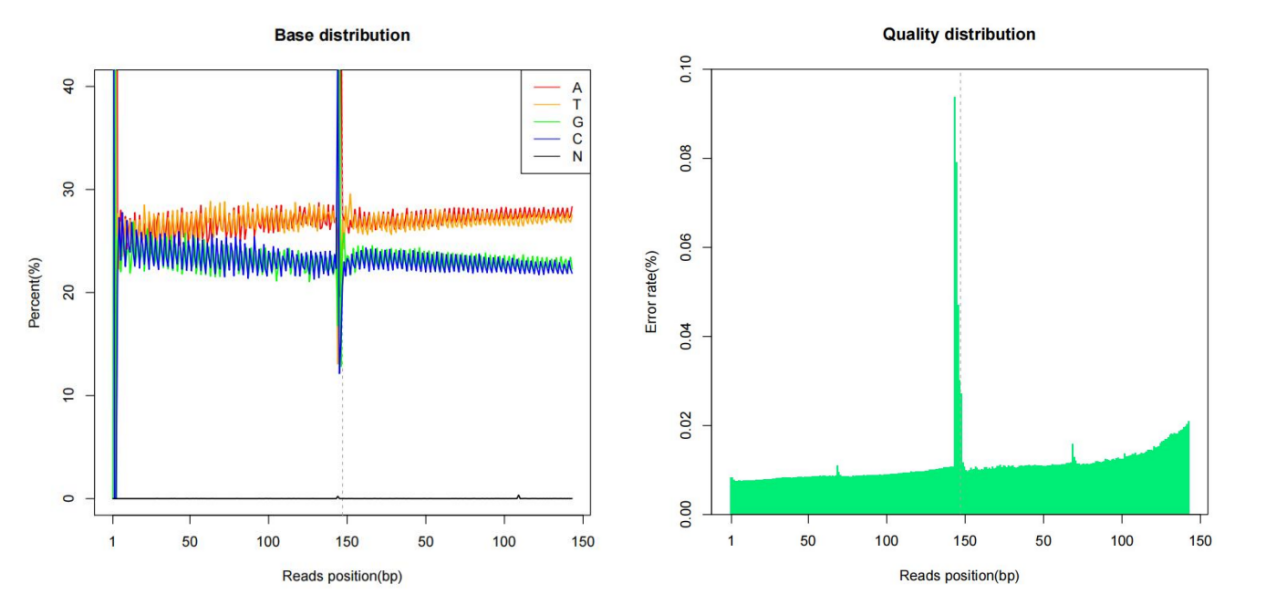

Supplement: S1 Fig — (TIF) [file pone.0324161.s001.tif]
