# Supplementary material for: Population genetic diversity and structure of the endangered species Tetracentron sinense Oliver (Tetracentraceae) with SNPs based on RAD sequencing
Source: PLoS One. 2025 May 20;20(5):e0324161. doi: 10.1371/journal.pone.0324161 (PMC12091802; doi:10.1371/journal.pone.0324161)
Supplement: S2 Fig — (PDF) [file pone.0324161.s002.pdf]

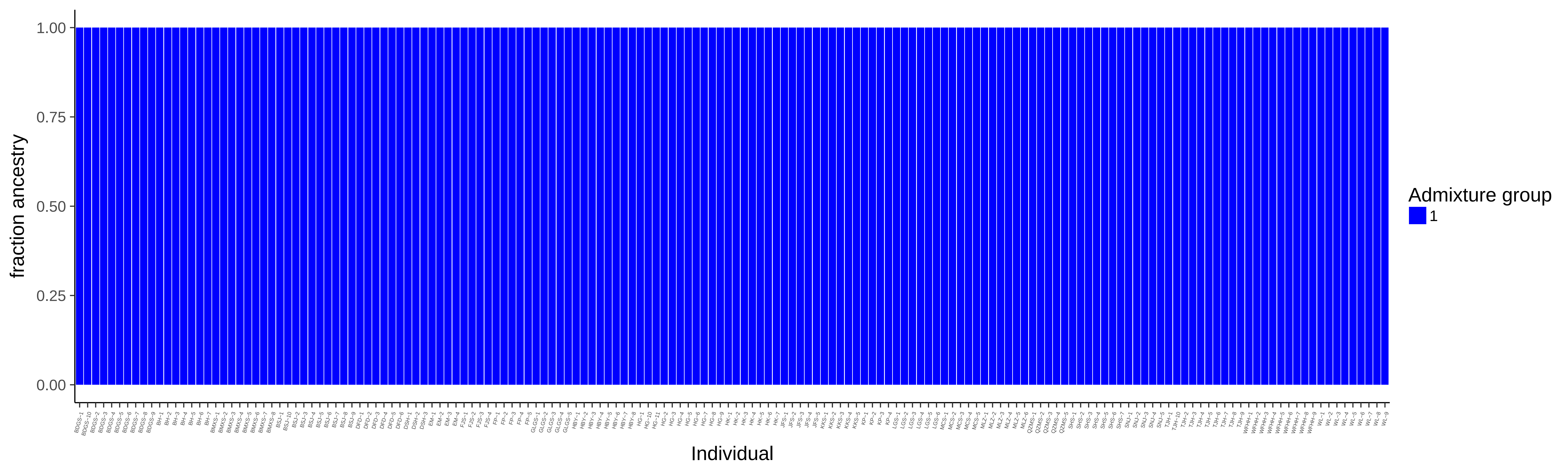
fraction ancestry
1.00
0.75
0.50
0.25
0.00
Individual
Admixture group
1

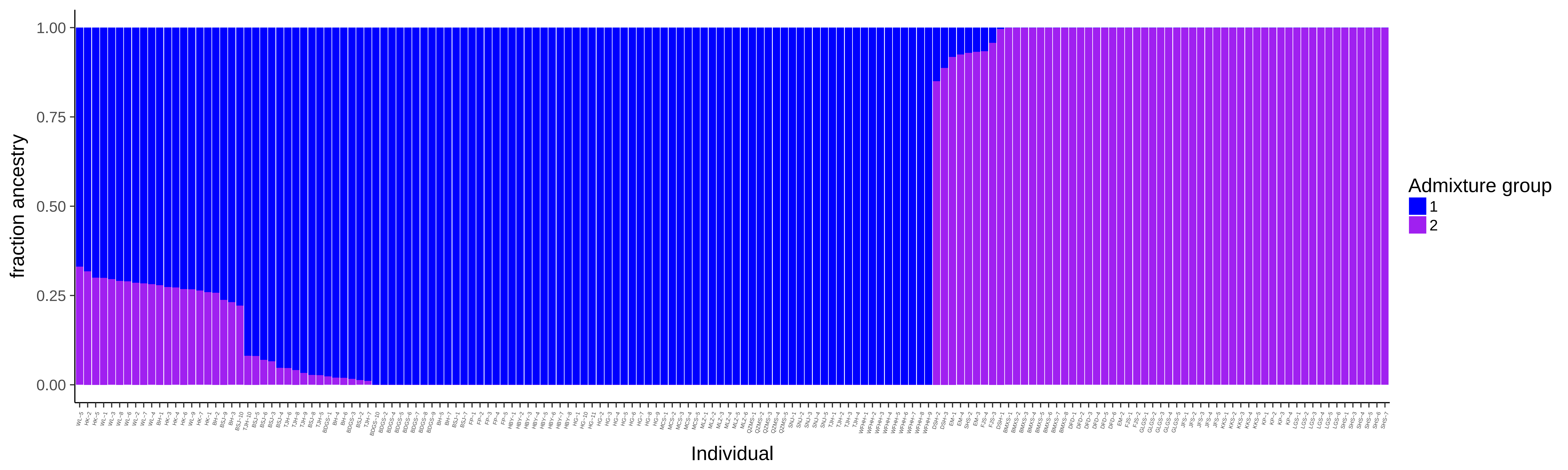
fraction ancestry
1.00
0.75
0.50
0.25
0.00
Individual
Admixture group
1
2

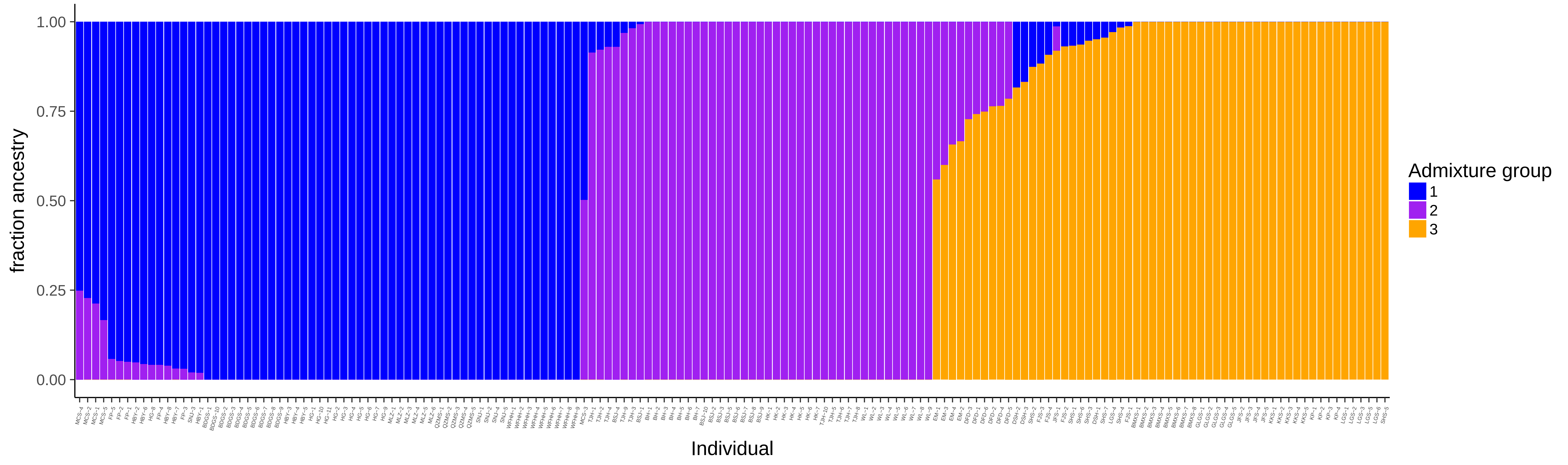

1.00
0.75
0.50
0.25
0.00
fraction ancestry
Individual
Admixture group
1
2
3

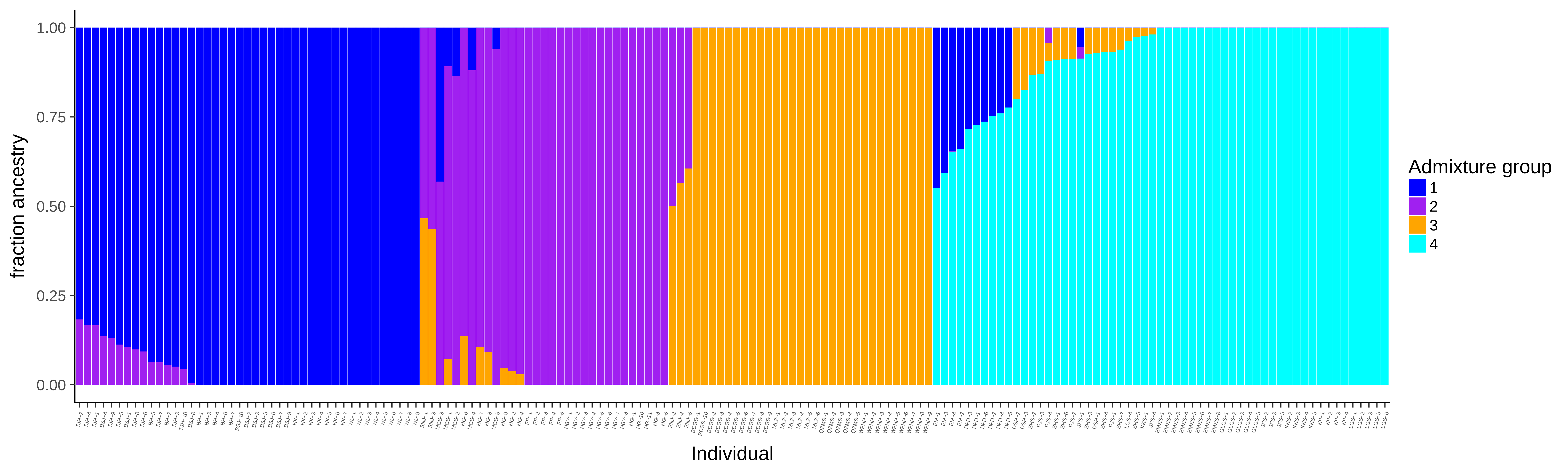
1.00
0.75
0.50
0.25
0.00
fraction ancestry
Individual
Admixture group
1
2
3
4

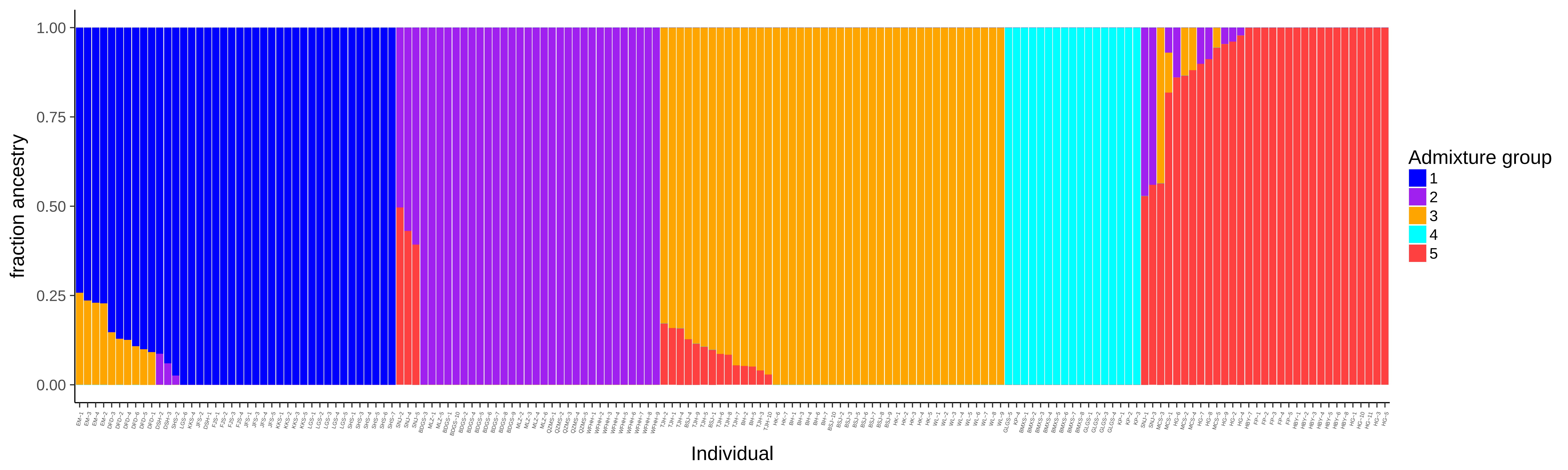
fraction ancestry
1.00
0.75
0.50
0.25
0.00
Individual
Admixture group
1
2
3
4
5

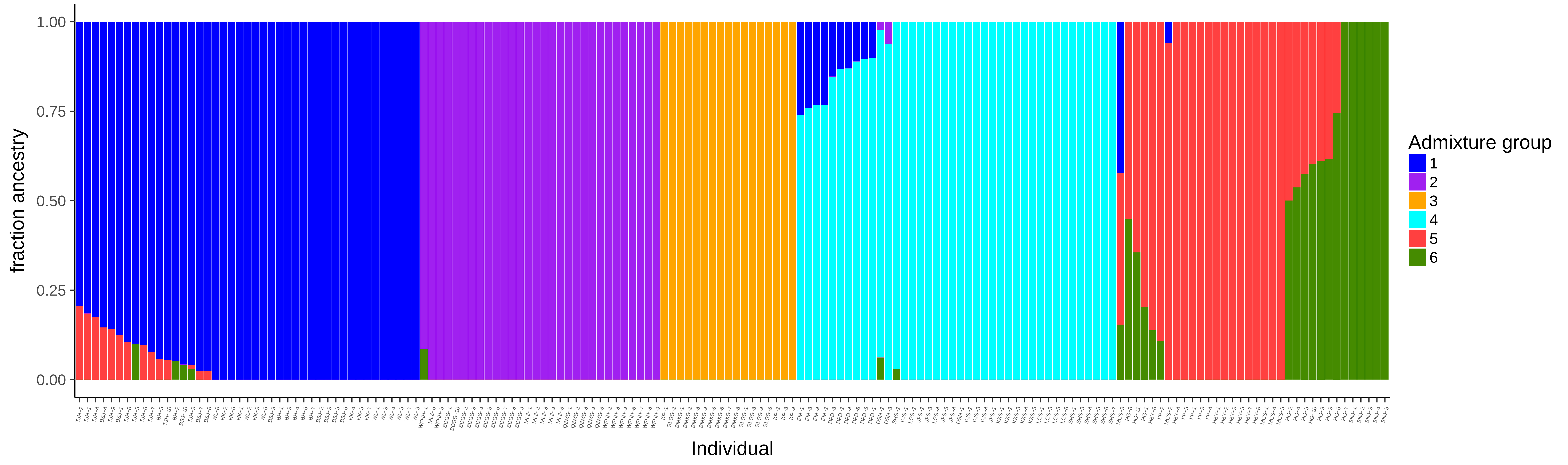
1.00
0.75
0.50
0.25
0.00
fraction ancestry
Individual
Admixture group
1
2
3
4
5
6

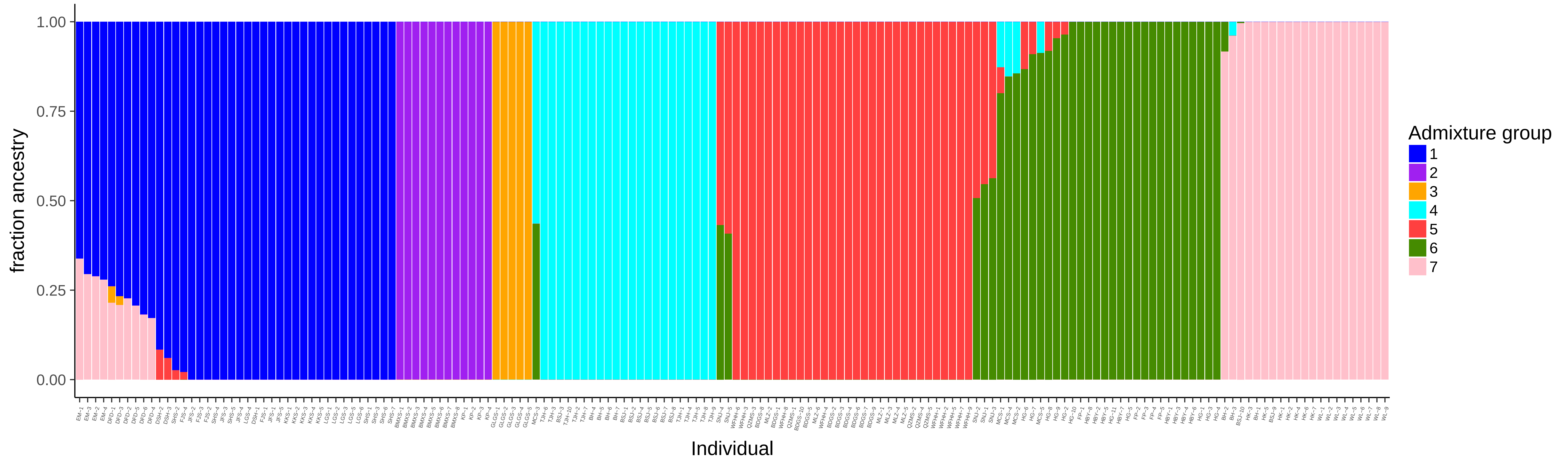
fraction ancestry
1.00
0.75
0.50
0.25
0.00
Individual
Admixture group
1
2
3
4
5
6
7

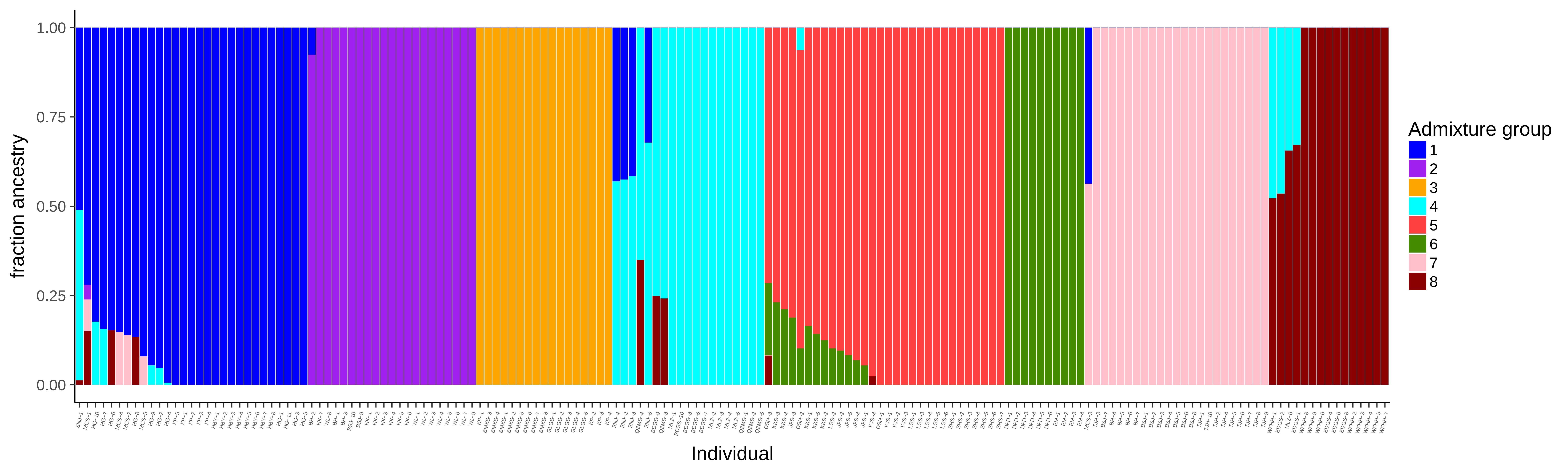

fraction ancestry
1.00
0.75
0.50
0.25
0.00
Individual
Admixture group
1
2
3
4
5
6
7
8

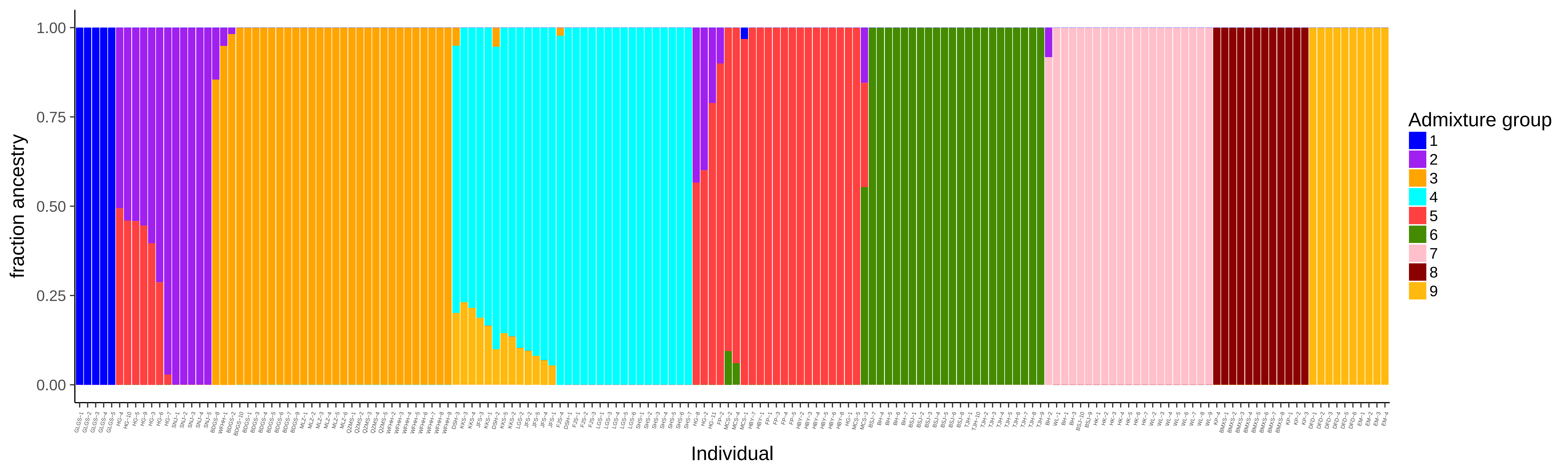
1.00
0.75
0.50
0.25
0.00
fraction ancestry
Individual
Admixture group
1
2
3
4
5
6
7
8
9

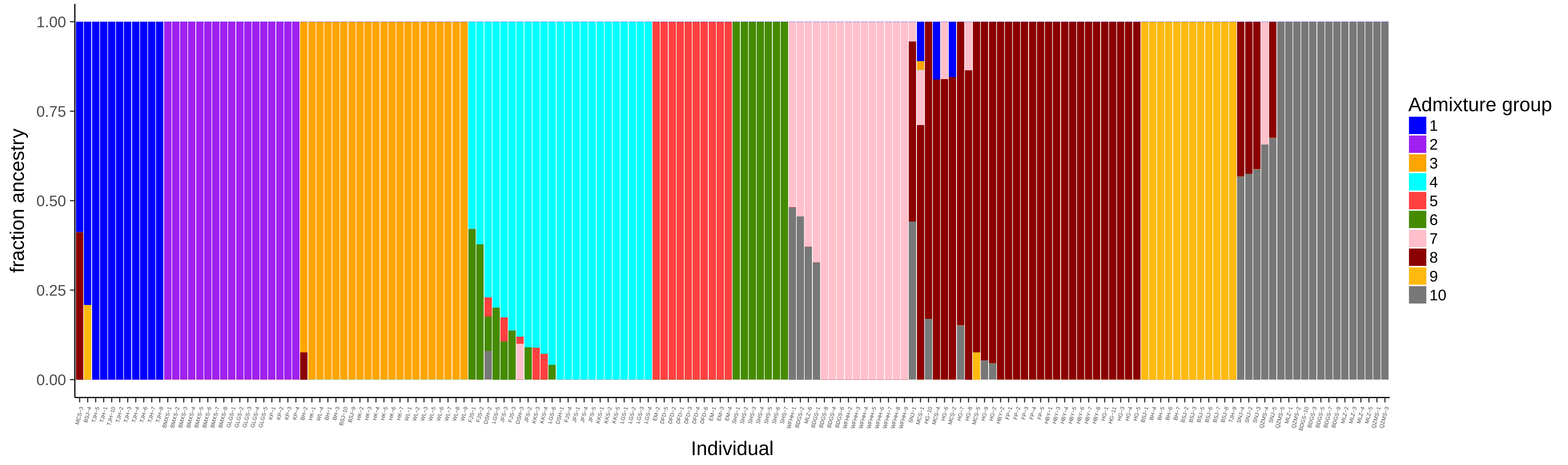
fraction ancestry
1.00
0.75
0.50
0.25
0.00
Individual
Admixture group
1
2
3
4
5
6
7
8
9
10

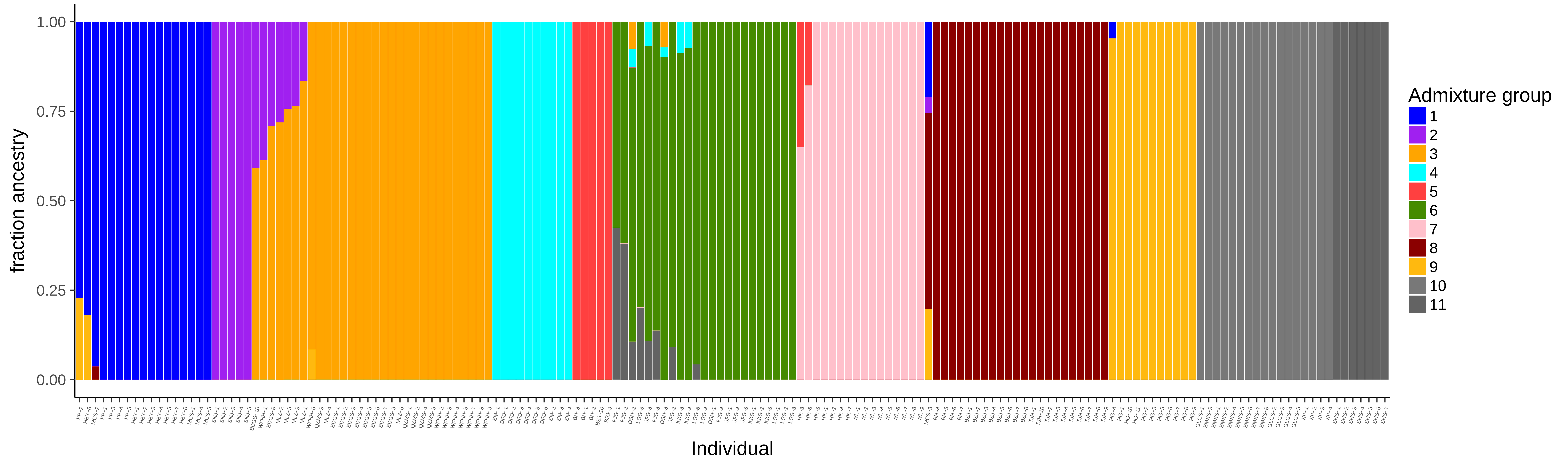
fraction ancestry
1.00
0.75
0.50
0.25
0.00
Individual
Admixture group
1
2
3
4
5
6
7
8
9
10
11

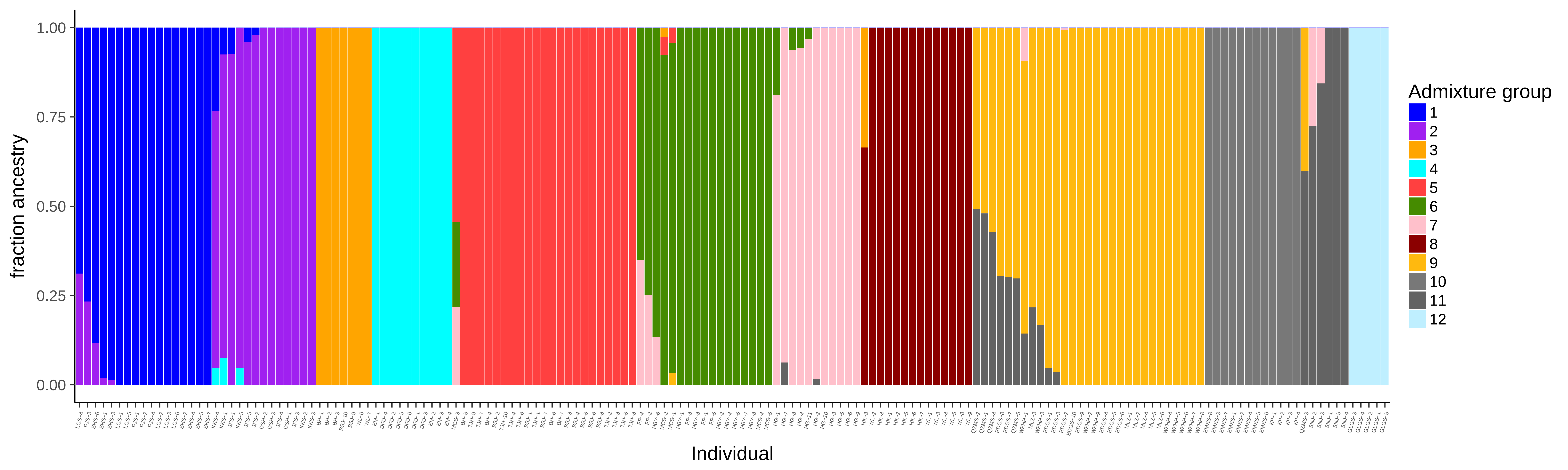

fraction ancestry
1.00
0.75
0.50
0.25
0.00
Individual
Admixture group
1
2
3
4
5
6
7
8
9
10
11
12

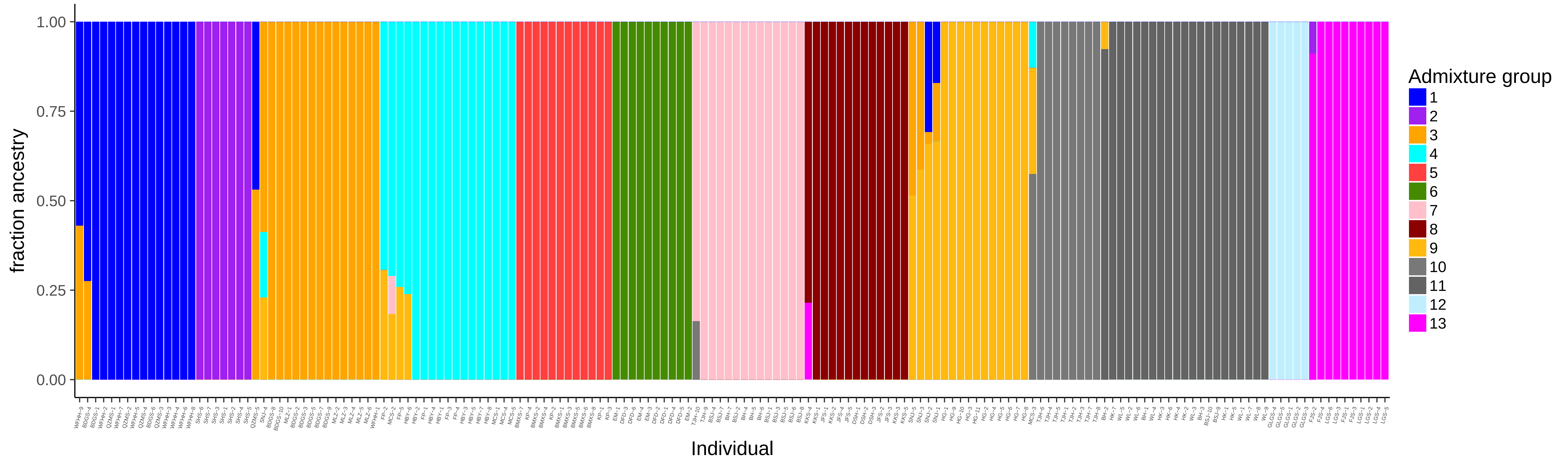
fraction ancestry
1.00
0.75
0.50
0.25
0.00
Individual
Admixture group
1
2
3
4
5
6
7
8
9
10
11
12
13

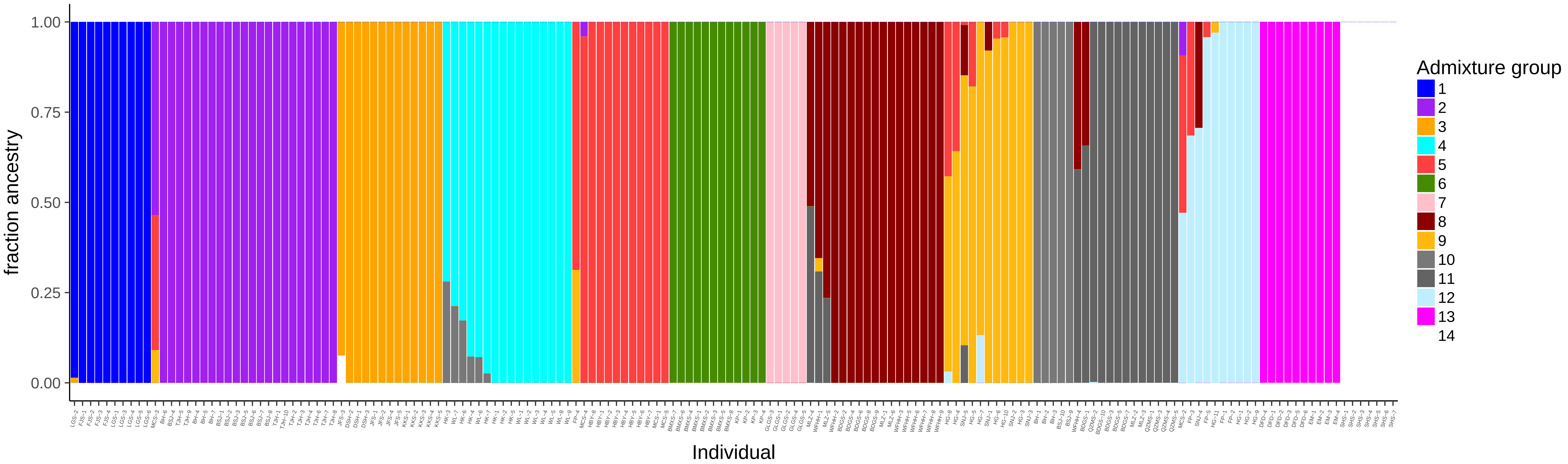
fraction ancestry
1.00
0.75
0.50
0.25
0.00
Individual
Admixture group
1
2
3
4
5
6
7
8
9
10
11
12
13
14

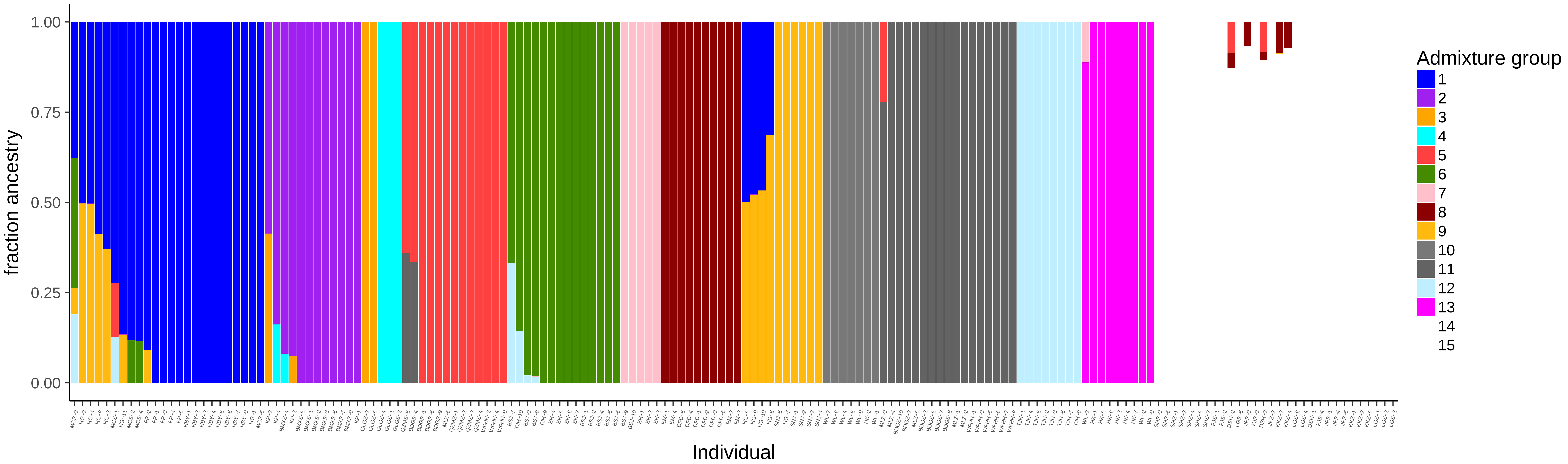

1.00
0.75
0.50
0.25
0.00
fraction ancestry
Individual
Admixture group
1
2
3
4
5
6
7
8
9
10
11
12
13
14
15

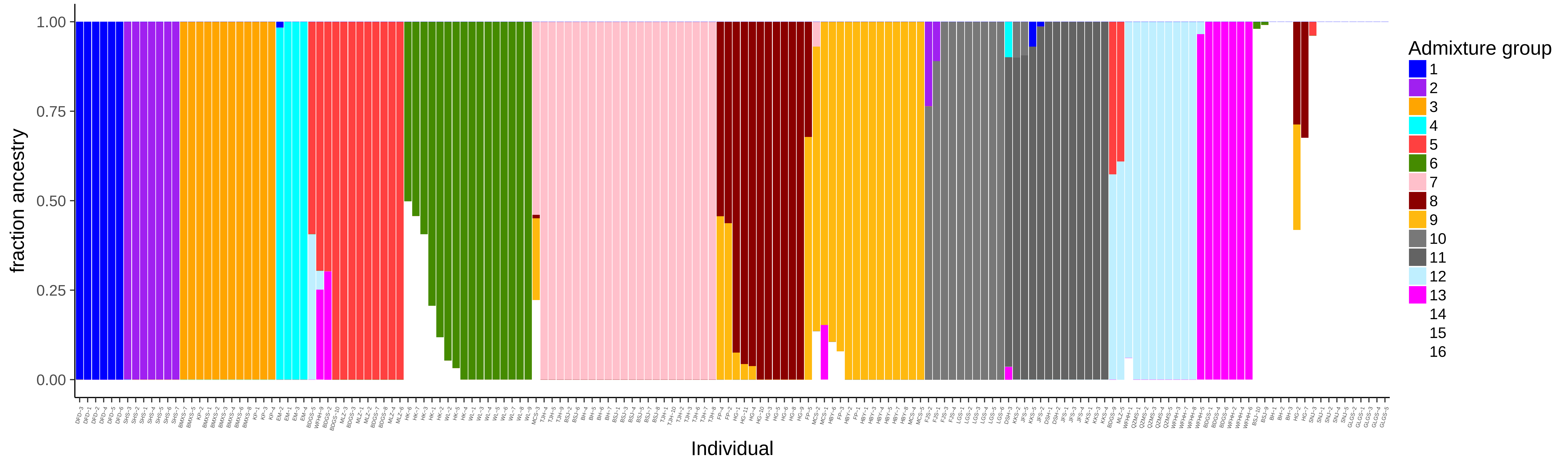
fraction ancestry
1.00
0.75
0.50
0.25
0.00
Individual
Admixture group
1
2
3
4
5
6
7
8
9
10
11
12
13
14
15
16

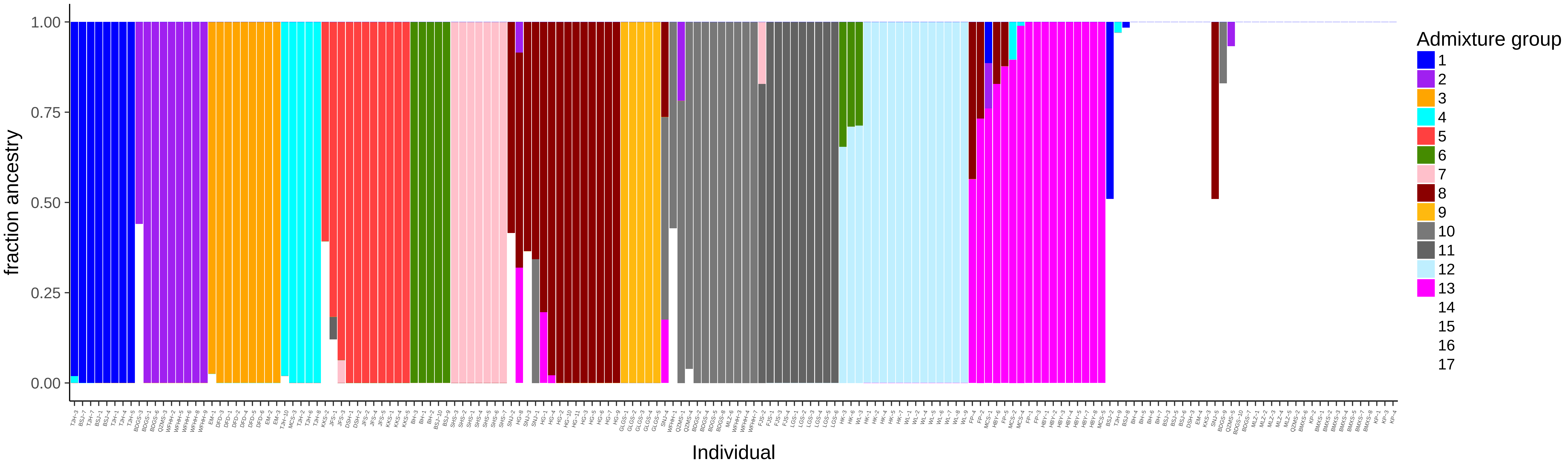

fraction ancestry
1.00
0.75
0.50
0.25
0.00
Individual
Admixture group
1
2
3
4
5
6
7
8
9
10
11
12
13
14
15
16
17

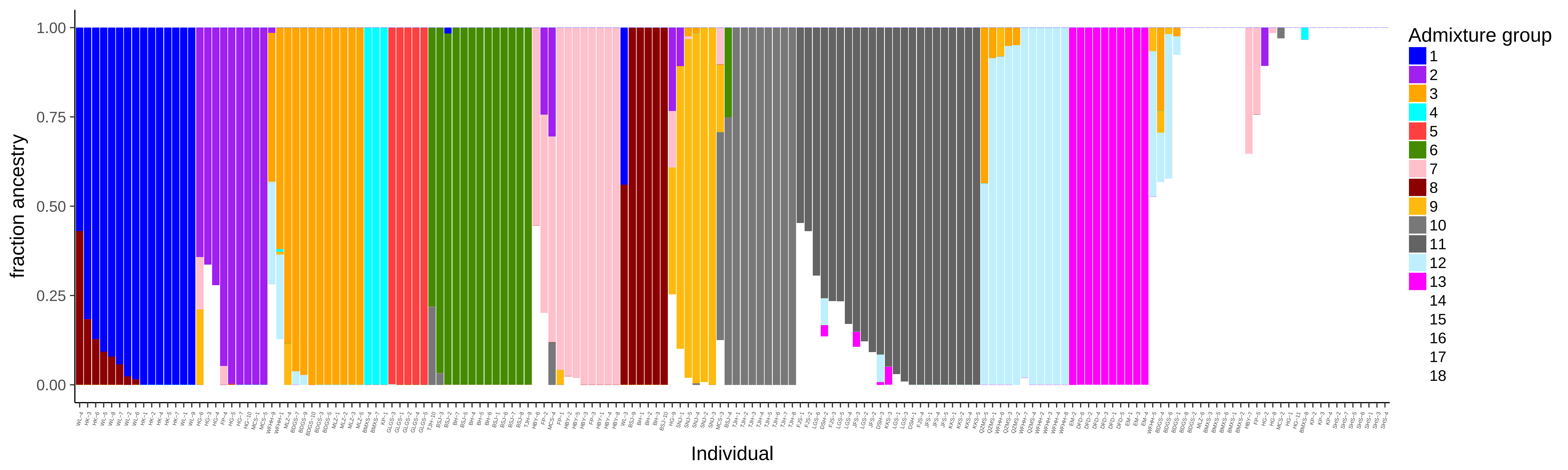
fraction ancestry
1.00
0.75
0.50
0.25
0.00
Individual
Admixture group
1
2
3
4
5
6
7
8
9
10
11
12
13
14
15
16
17
18

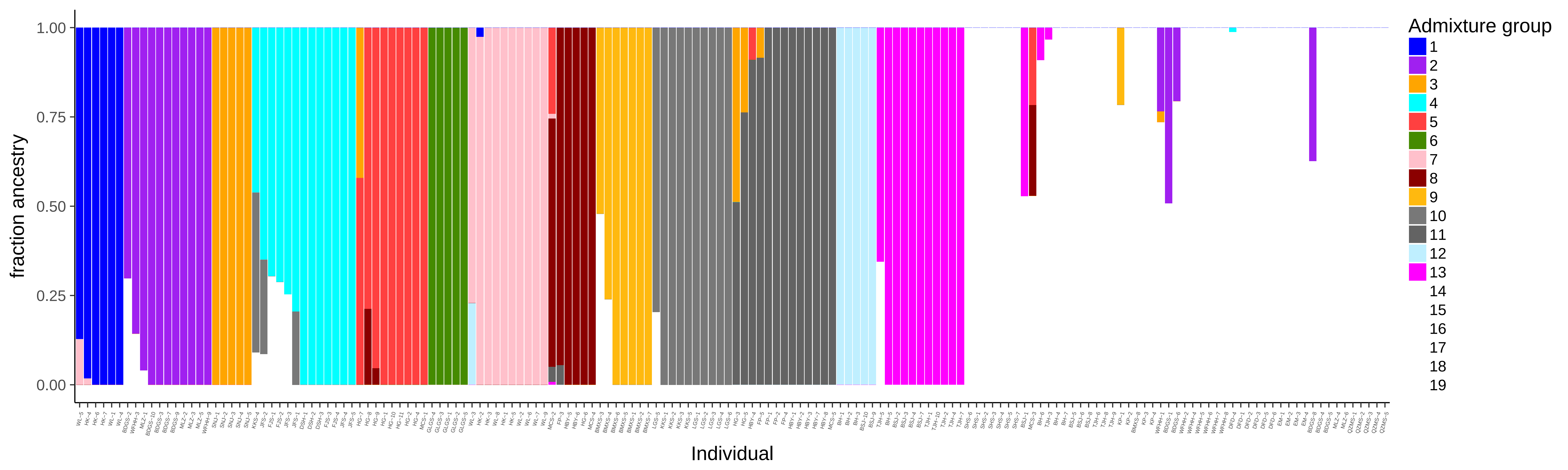

fraction ancestry
1.00
0.75
0.50
0.25
0.00
Individual
Admixture group
1
2
3
4
5
6
7
8
9
10
11
12
13
14
15
16
17
18
19

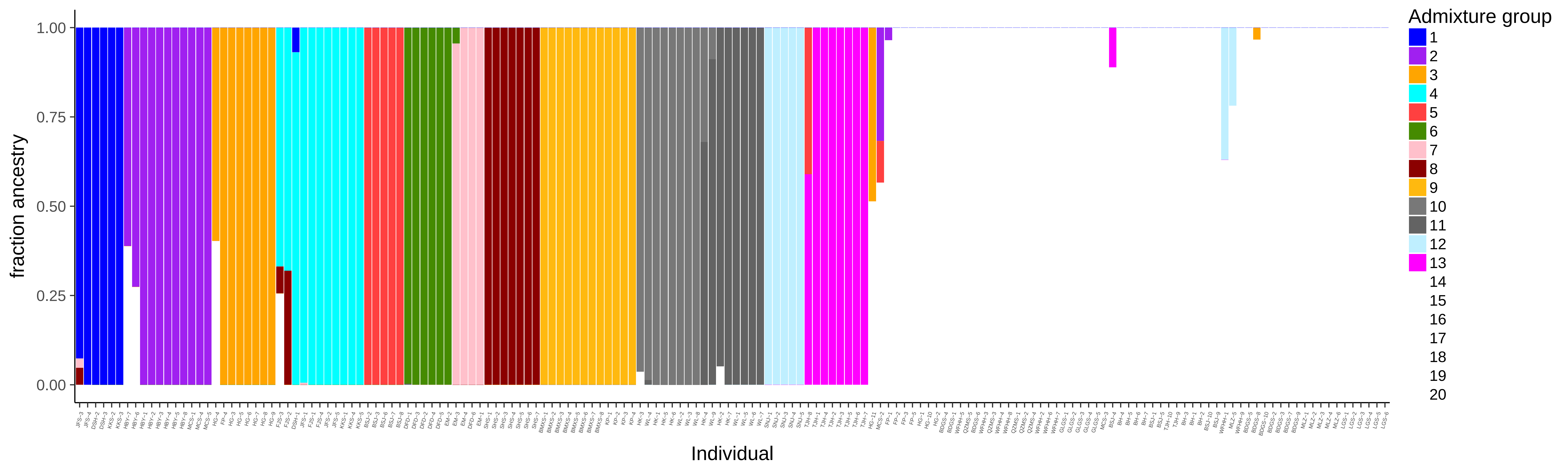
fraction ancestry
1.00
0.75
0.50
0.25
0.00
Individual
Admixture group
1
2
3
4
5
6
7
8
9
10
11
12
13
14
15
16
17
18
19
20

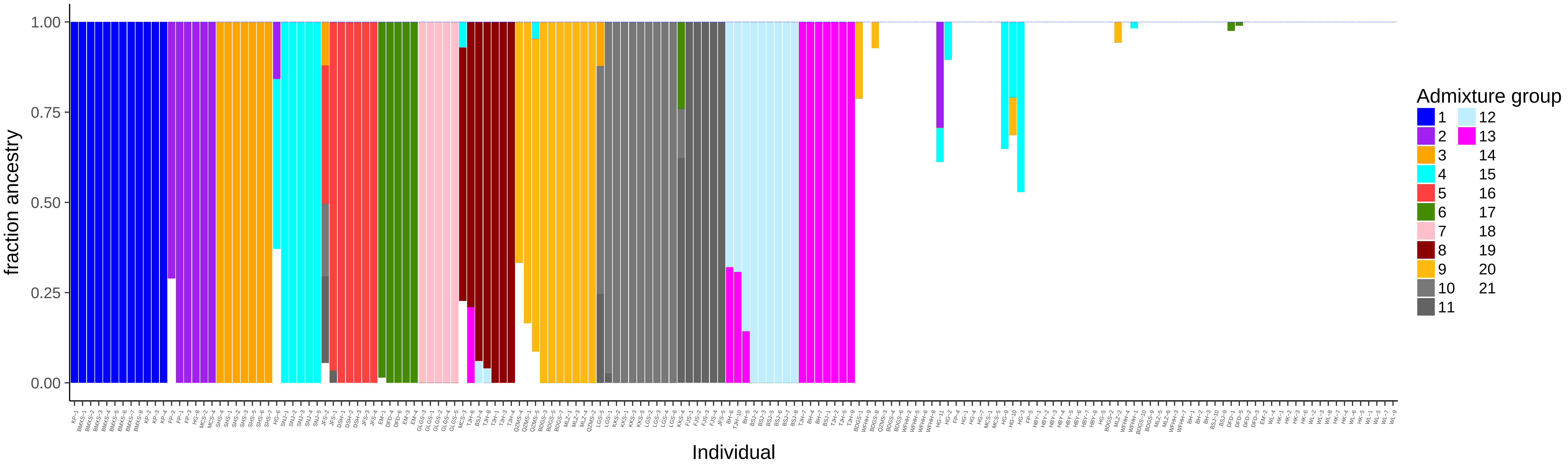
fraction ancestry
1.00
0.75
0.50
0.25
0.00
Individual
Admixture group
1
2
3
4
5
6
7
8
9
10
11
12
13
14
15
16
17
18
19
20
21

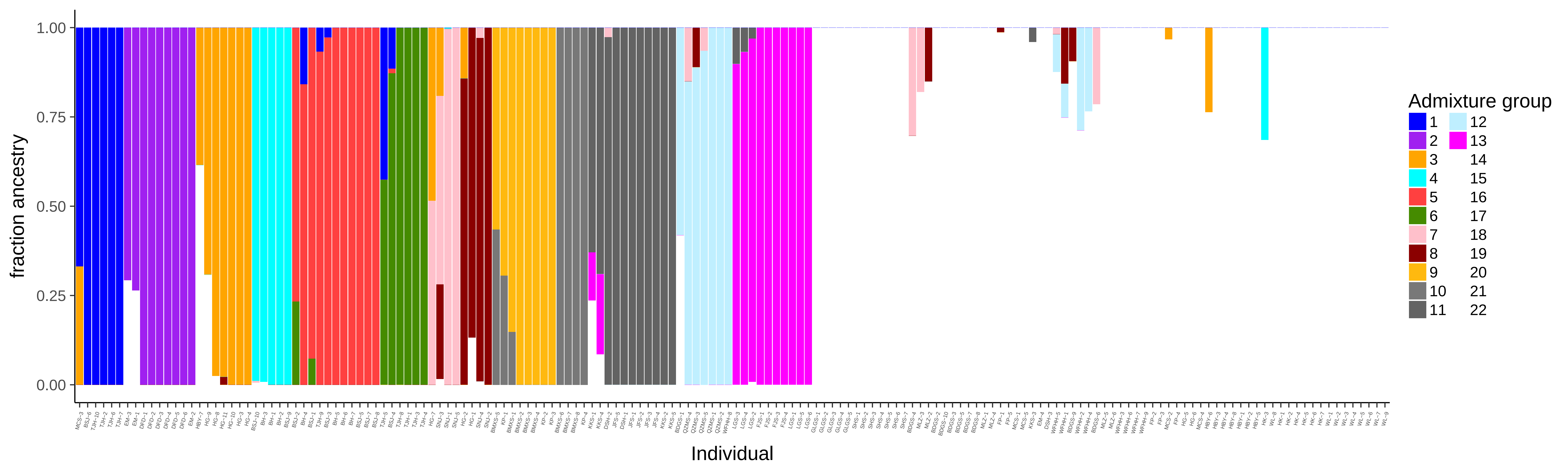
1.00
0.75
0.50
0.25
0.00
fraction ancestry
Individual
Admixture group
1
2
3
4
5
6
7
8
9
10
11
12
13
14
15
16
17
18
19
20
21
22

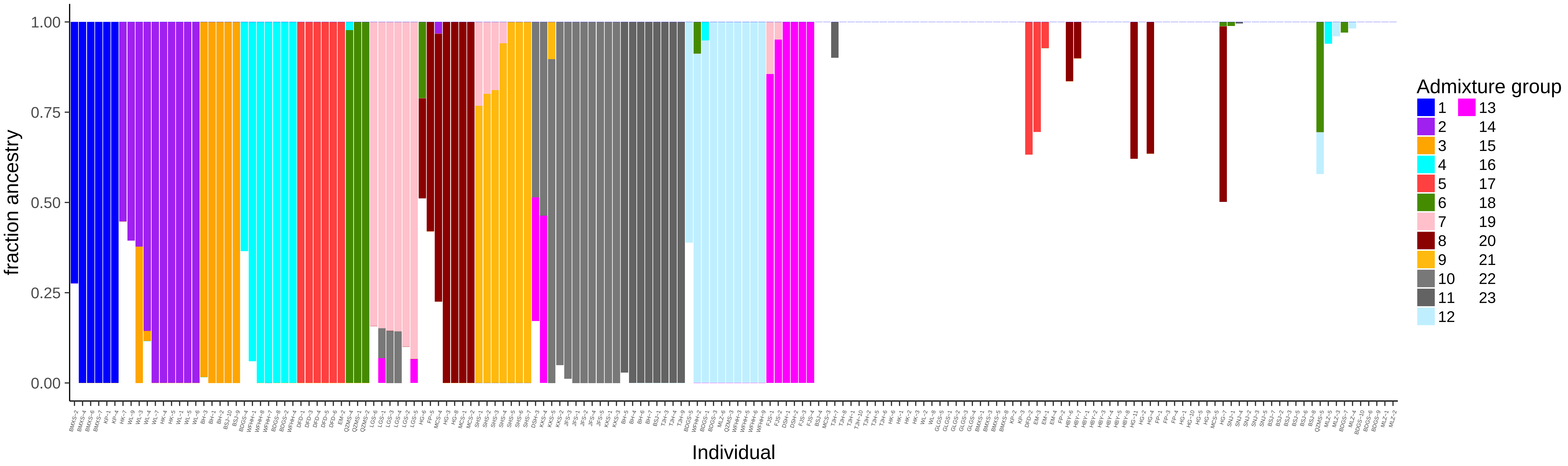
fraction ancestry
1.00
0.75
0.50
0.25
0.00
Individual
Admixture group
1
2
3
4
5
6
7
8
9
10
11
12
13
14
15
16
17
18
19
20
21
22
23

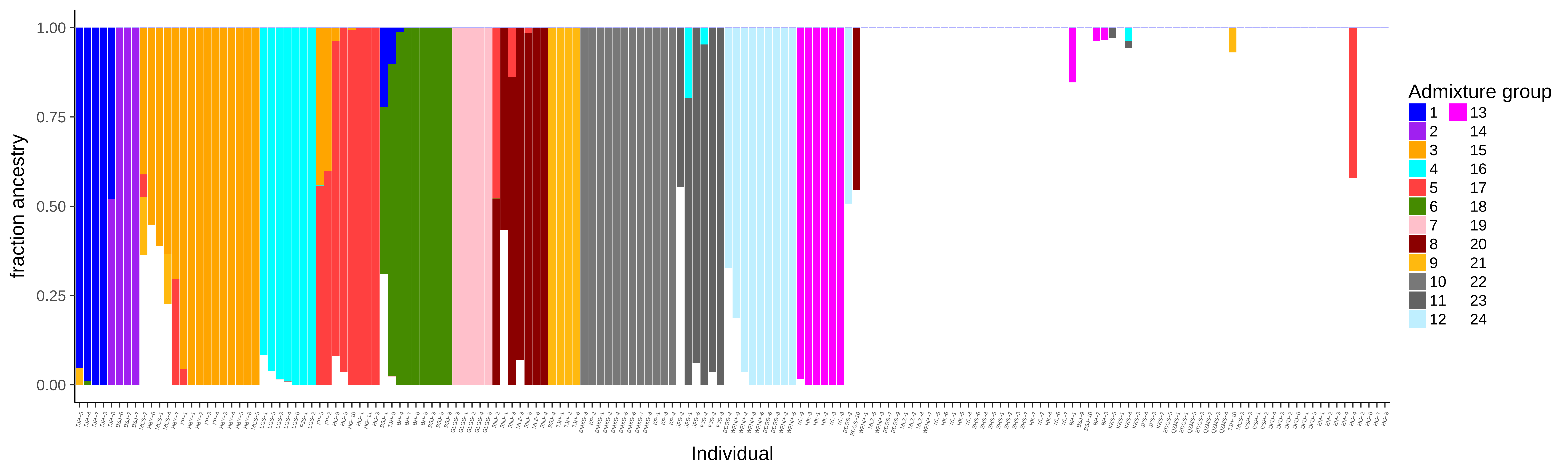
1.00
0.75
0.50
0.25
0.00
fraction ancestry
Individual
Admixture group
1
2
3
4
5
6
7
8
9
10
11
12
13
14
15
16
17
18
19
20
21
22
23
24

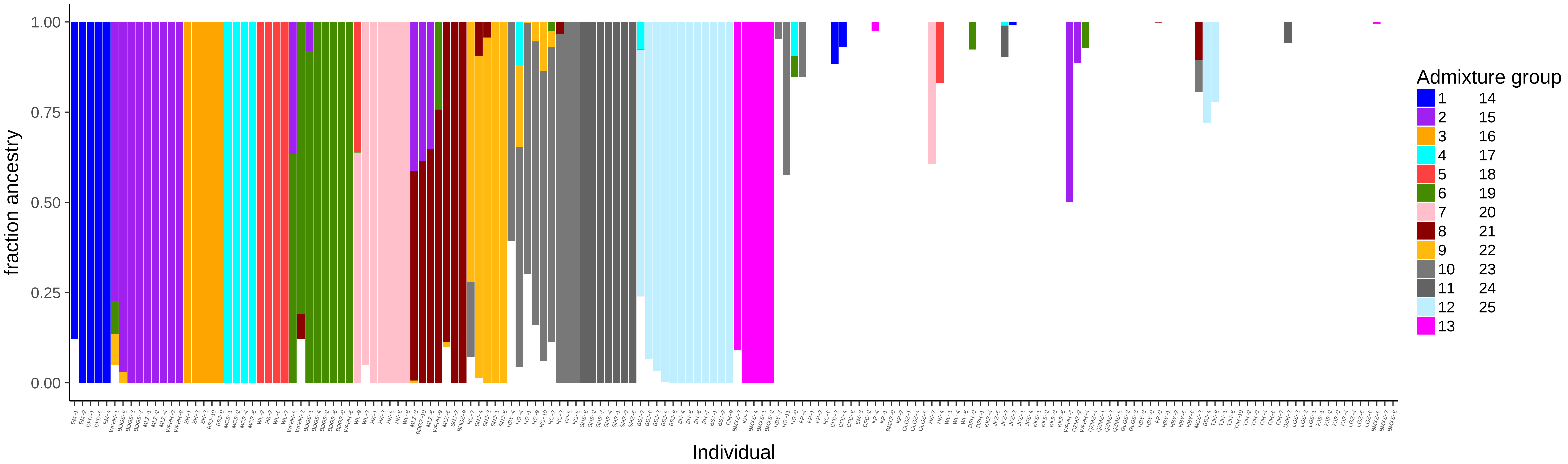
fraction ancestry
1.00
0.75
0.50
0.25
0.00
Individual
Admixture group
1
2
3
4
5
6
7
8
9
10
11
12
13
14
15
16
17
18
19
20
21
22
23
24
25

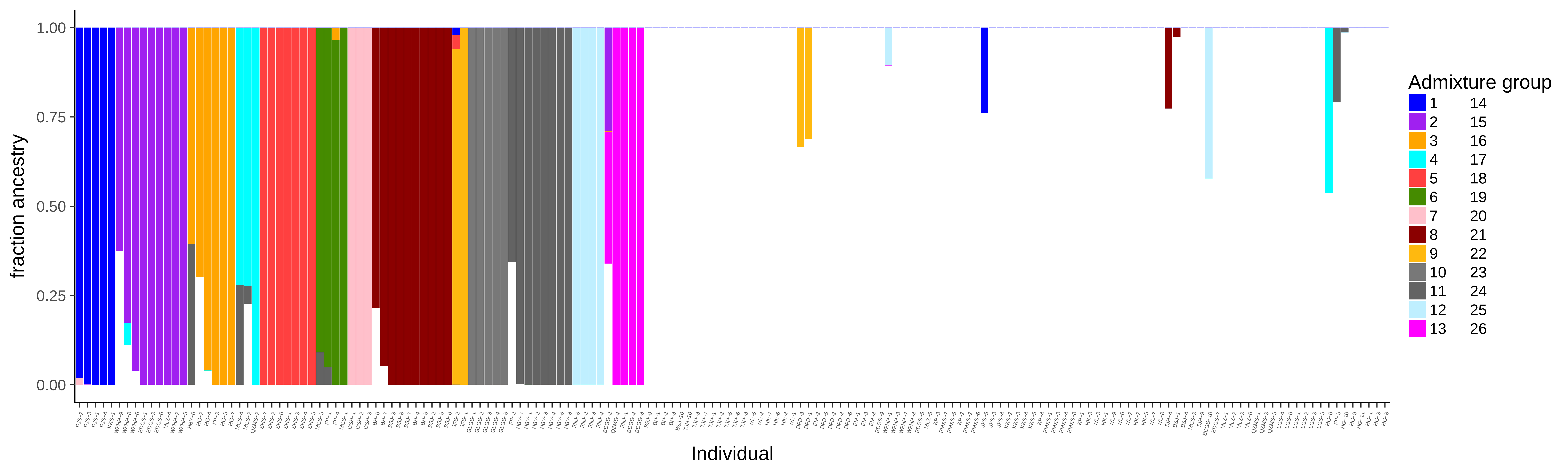
fraction ancestry
1.00
0.75
0.50
0.25
0.00
Individual
Admixture group
1
2
3
4
5
6
7
8
9
10
11
12
13
14
15
16
17
18
19
20
21
22
23
24
25
26

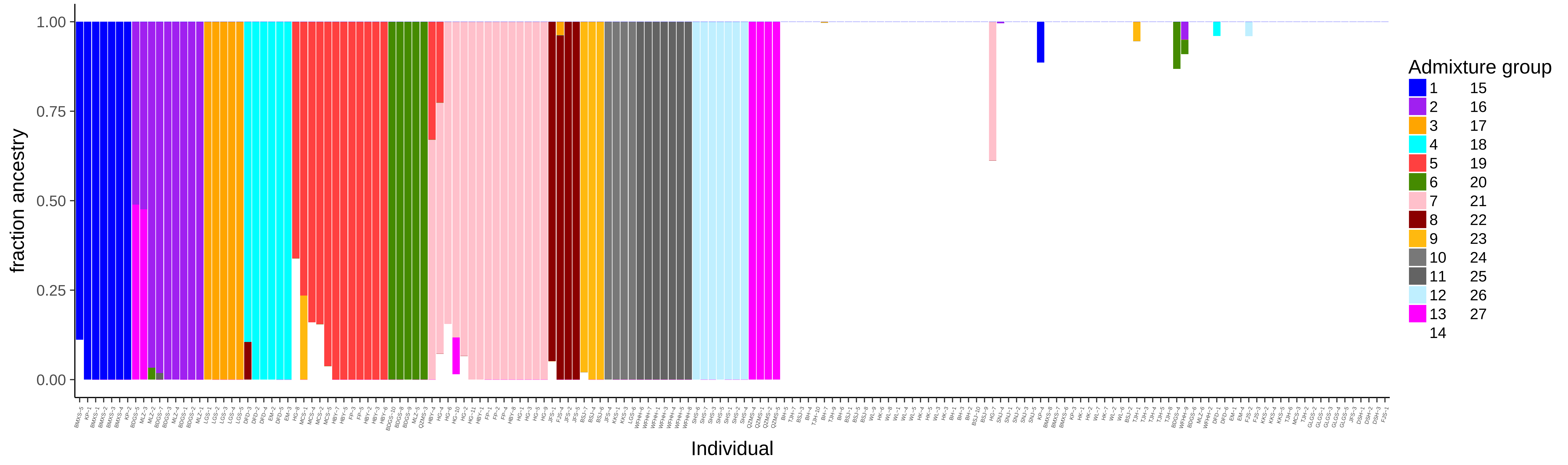

fraction ancestry
1.00
0.75
0.50
0.25
0.00
Individual
Admixture group
1
2
3
4
5
6
7
8
9
10
11
12
13
14
15
16
17
18
19
20
21
22
23
24
25
26
27
