# Supplementary material for: Population genetic diversity and structure of the endangered species Tetracentron sinense Oliver (Tetracentraceae) with SNPs based on RAD sequencing
Source: PLoS One. 2025 May 20;20(5):e0324161. doi: 10.1371/journal.pone.0324161 (PMC12091802; doi:10.1371/journal.pone.0324161)
Supplement: Attachment — Submitted filename: PLOS-review.docx [file pone.0324161.s010.docx]

**Population genetic diversity and structure of the endangered species *Tetracentron***

***sinense* Oliver (Tetracentraceae) with SNPs based on RAD sequencing.**

**Abstract**

Line 25 to 28- The sentence is long and confusing. Break into two shorter clearer sentences.

Line 32 to 37 – Edit to remove numbers (1), (2) and (3).

Enrich the results section by adding in information on the additional results from the recommended analysis.

Line 38- Delete the word ‘Finally’.

Line 40 – Break the sentence into two so that the first sentence ends at geographic groups. Delete ‘and that'. Start new sentence with ‘Attention’.

Align the conclusion with the additional recommended analyses.

**Introduction**

A relevant introduction has been provided however the grammar needs to be improved. Some of the changes to be made are listed below:

Line 48 delete the word ‘very’

Line 51 delete the words 'endangered species particularly’. Replace ‘cause’ with causes.

Line 52 replace the word with ‘affect’ with affects.

Line 56 – delete the word ‘great’.

Line 57 delete ‘a’ before small

Line 59 italicise “Tetracentron”

Line 62 edit sentence to remove the abbreviation e.g.

Lien 67 add the words ‘have been done’ after techniques

Line 83 to 87 The sentence is long and confusing. Break into two shorter clearer sentences.

Line 96 edit last part of the sentence to read ‘low cost with high reliability’

**Materials and methods**

Appropriate methods were used. The section needs to be improved as detailed below.

In the methodology section, line 107-108 the statement “Samples of spaced about 50m apart were randomly selected…” does not make grammatical sense. What did the authors mean?

Line 131: It would be good to declare the type of alignment done after running the process_radtags programme. The authors are leaving the readers to assume that a *de novo* assembly was done based on the flow from ustacks to cstacks and sstacks.

Although its mentioned in the methodology as a filtering parameter, the proportion of SNPs with a minor allele frequency (MAF) ≥ 0.05 is not declared in the results. There is need to declare the proportion of these SNPs before and after filtering.

Population stratification has been explored. However, more analysis should be done to implore the spatial population structure using TESS (refer to Front Genet 2019 10:954, Fig. 1).

Additional aspects of genetic diversity should be explored. Some important analysis such as linkage disequilibrium (LD) patterns and samples’ co-ancestry have not been done. The authors are encouraged to explore this option since it would provide information on the historical recombinations that could have occurred within samples as well as help in understanding the observed population structure.

The authors are also encouraged to explore using OutFLANK and PCAdapt approaches to identify the putative adaptive loci as well as the outlier SNPs.

Editing to remove grammatical errors to be done in whole section.

**Results**

Commendable effort has been made to report the results. The manuscript can be improved as follows.

It is recommended that the authors compute and include in the results section other genetic diversity indices such as the Hardy-Weinberg Equilibrium (HWE) test scores for the different *T. sinense* populations under study.

There are certain important summary statistics that could be included in the analysis such as the Watterson’s theta which would also help in understanding the TajimaD statistics.

Important population parameters such as site frequency spectra (SFS) to show the distribution of alleles in the different populations of *T. sinense* also need to be provided. This is important because it groups the SNPs into classes based on how many copies of the minor allele they possess and then calculates the size of each group. As such, this will provide the readers with information on the distribution of alleles in the different populations under study.

Results from the additional analyses should be inserted in the appropriate sections of the results.

Editing to remove grammatical errors will improve the section.

**Discussion and conclusions**

The authors have tried to discuss the results described in the manuscript. The discussion can be further improved.

Align the discussion and conclusions with the results from the recommended additional analysis.

The sections has too many grammar errors to enumerate. This needs to be diligently addressed.
