# Supplementary material for: Population genetic diversity and structure of the endangered species Tetracentron sinense Oliver (Tetracentraceae) with SNPs based on RAD sequencing
Source: PLoS One. 2025 May 20;20(5):e0324161. doi: 10.1371/journal.pone.0324161 (PMC12091802; doi:10.1371/journal.pone.0324161)
Supplement: Attachment — Submitted filename: Response to Reviewers.docx [file pone.0324161.s013.docx]

**Author’s Response to reviewers’ Comments**

Dear editor,

Thank you very much for giving us an opportunity to revise our manuscript. We appreciate the reviewers very much for their constructive comments and suggestions on our manuscript entitled “**Population genetic diversity and structure of the endangered species *Tetracentron sinense* Oliver ([Tetracentraceae](http://www.iplant.cn/info/Tetracentraceae)) with SNPs based on RAD sequencing**”.

We have studied reviewers’ comments carefully. According to the reviewers’ detailed suggestions, we have made a careful revision on the original manuscript.

Kind regards.

Zhong–Qiong Tian

E–mail: tzq@utibet.edu.cn

Corresponding author: Xiao–Hong Gan

E–mail : [bhgan@cwnu.edu.cn](mailto:bhgan@cwnu.edu.cn)

**Replies to the reviewers’ comments:** The main corrections are in the manuscript and the responds to the reviewers’ comments are as follows (the replies are highlighted in blue).

Line 25 to 28- The sentence is long and confusing. Break into two shorter clearer sentences.

Response: We have breaked the long sentence into two shorter clearer sentences with the content of– As a tertiary living fossil, a dramatic decline in *Tetracentron sinense* Oliv. population amounts. Nowadays, genetic resource depletion and human activities have shaped habitat fragmentation of relict and endangered plants, although there is ample evidence of its great medicinal, economic and ecological value;

Enrich the results section by adding in information on the additional results from the recommended analysis.

Response: we have enriched the results part;

Line 38- Delete the word ‘Finally’.

Response: We have deleted the word ‘Finally’;

Line 40 – Break the sentence into two so that the first sentence ends at geographic groups. Delete ‘and that'. Start new sentence with ‘Attention’.

Response: We have breaked the long sentence into two shorter clearer sentences with the content of–For the preservation of the genetic integrity of *Tetracentron sinense* Oliv., we suggest that conservation units should be established corresponding to different geographic groups. Attention should be paid to low/high genetic diversity populations, such as establishing artificial reproduction and germplasm resource nurseries;

A relevant introduction has been provided however the grammar needs to be improved. Some of the changes to be made are listed below:

Line 48 delete the word ‘very’

Line 51 delete the words 'endangered species particularly’. Replace ‘cause’ with causes.

Line 52 replace the word with ‘affect’ with affects.

Line 56 – delete the word ‘great’.

Line 57 delete ‘a’ before small

Line 59 italicise “Tetracentron”

Line 62 edit sentence to remove the abbreviation e.g.

Lien 67 add the words ‘have been done’ after techniques

Response: The content of line 48-67 in our study is corrected according to the requirements of the reviewer;

Line 83 to 87 The sentence is long and confusing. Break into two shorter clearer sentences.

Response: We have breaked the long sentence into two shorter clearer sentences with the content of–Gan et al. (2013) believed that the small population size limited the efficiency of insect and wind pollination during flowering and pollination (the rainy season) of *T. sinense*, resulting in reduced genetic diversity and high genetic differentiation within the population, and the geographical isolation among populations was a factor restricting gene flow, which might be a crucial factor leading to the endangerment [13];

Line 96 edit last part of the sentence to read ‘low cost with high reliability’

Response: is relatively low cost and the results are reliable;

Appropriate methods were used. The section needs to be improved as detailed below.

In the methodology section, line 107-108 the statement “Samples of spaced about 50m apart were randomly selected…” does not make grammatical sense. What did the authors mean?

Response: We eadjust the sentence content to–Samples with a spatial distance of more than 50 meters were selected to avoid the close relationship between individuals, and leaf materials were collected into silica gel for drying and preservation;

Additional aspects of genetic diversity should be explored. Some important analysis such as linkage disequilibrium (LD) patterns and samples’ co-ancestry have not been done. The authors are encouraged to explore this option since it would provide information on the historical recombinations that could have occurred within samples as well as help in understanding the observed population structure.

Response: We added the PopLDdecay analyses in our study;

The grammar of this manuscript has been carefully checked and revised.
