# Supplementary material for: Population genetic diversity and structure of the endangered species Tetracentron sinense Oliver (Tetracentraceae) with SNPs based on RAD sequencing
Source: PLoS One. 2025 May 20;20(5):e0324161. doi: 10.1371/journal.pone.0324161 (PMC12091802; doi:10.1371/journal.pone.0324161)
Supplement: Attachment — Submitted filename: Revision Comments.docx [file pone.0324161.s012.docx]

**Dear Editor**

Overall, the authors have made good effort to address the concerns we raised on the previous manuscript draft. I draw your attention to the following:

1. Methodology: Line 128, The authors should declare explicitly that they did a *de novo* assembly for the benefit of the readers who are not familiar with the flow of events in stacks software. This had been raised earlier
2. Using the GPS coordinates of the sampling sites together with the SNP data, the authors could explore ancestry estimation for spatial population genetic analysis using TESS as earlier suggested. This will enable the evaluation on whether *T. sinense* populations in central and south China have distinct or mixed origins and enrich the ADMIXTURE results. The generated map(s) can then be added to the results.
3. Results: It is commendable that the authors explored the linkage disequilibrium (LD) pattern of the SNP markers in the population. However, an LD scatter plot would be more informative to show the decay rate as explained in lines 251-257. I suggest replacing Table 6 with an LD scatter plot.
4. The grammar and writing style also improved. However, the final draft will need proof reading and we recommend that the authors get an English-speaking colleague to check for grammar and language use.

The items raised above can be checked by the editorial team and we don’t need to see the next draft.

Thank you for the opportunity to review.

Regards
