# Supplementary material for: Population genetic diversity and structure of the endangered species Tetracentron sinense Oliver (Tetracentraceae) with SNPs based on RAD sequencing
Source: PLoS One. 2025 May 20;20(5):e0324161. doi: 10.1371/journal.pone.0324161 (PMC12091802; doi:10.1371/journal.pone.0324161)
Supplement: Attachment — Submitted filename: Response_to_Reviewers_auresp_2.docx [file pone.0324161.s015.docx]

**Author’s Response to reviewers’ Comments**

Dear editor,

Thank you very much for giving us an opportunity to revise our manuscript. We appreciate the reviewers very much for their constructive comments and suggestions on our manuscript entitled “**Population genetic diversity and structure of the endangered species *Tetracentron sinense* Oliver ([Tetracentraceae](http://www.iplant.cn/info/Tetracentraceae)) with SNPs based on RAD sequencing**”.

We have studied reviewers’ comments carefully. According to the reviewers’ detailed suggestions, we have made a careful revision on the original manuscript.

Kind regards.

Zhong–Qiong Tian

E–mail: tzq@utibet.edu.cn

Corresponding author: Xiao–Hong Gan

E–mail : [bhgan@cwnu.edu.cn](mailto:bhgan@cwnu.edu.cn)

**Replies to the reviewers’ comments:** The main corrections are in the manuscript and the responds to the reviewers’ comments are as follows (the replies are highlighted in blue).

Line 25 to 28- The sentence is long and confusing. Break into two shorter clearer sentences.

Response: We have breaked the long sentence into two shorter clearer sentences with the content of– *Tetracentron sinense* Oliv. (*T. sinense*), as a tertiary living fossil, has experienced a significant decline in population numbers. Currently, genetic resources depletion and human activities have led to habitat fragmentation of relict and endangered plants, despite the abundant evidence of its medicinal, economic, and ecological value;

Enrich the results section by adding in information on the additional results from the recommended analysis.

Response: we have enriched the results part;

Line 38- Delete the word ‘Finally’.

Response: We have deleted the word ‘Finally’;

Line 40 – Break the sentence into two so that the first sentence ends at geographic groups. Delete ‘and that'. Start new sentence with ‘Attention’.

Response: We have breaked the long sentence into two shorter clearer sentences with the content of–To preserve the genetic integrity of *T. sinense*, it is recommended to establish conservation units corresponding to different geographic groups, with a focus on populations with low/high genetic diversity by implementing artificial reproduction and germplasm resource nurseries. Given the species’ vulnerable conservation status, urgent implementation of the aforementioned conservation strategies is necessary to safeguard the remaining genetic resources.

A relevant introduction has been provided however the grammar needs to be improved. Some of the changes to be made are listed below:

Line 48 delete the word ‘very’

Line 51 delete the words 'endangered species particularly’. Replace ‘cause’ with causes.

Line 52 replace the word with ‘affect’ with affects.

Line 56 – delete the word ‘great’.

Line 57 delete ‘a’ before small

Line 59 italicise “Tetracentron”

Line 62 edit sentence to remove the abbreviation e.g.

Lien 67 add the words ‘have been done’ after techniques

Response: The content of line 48-67 in our study is corrected according to the requirements of the reviewer;

Line 96 edit last part of the sentence to read ‘low cost with high reliability’

Response: is relatively low cost and the results are reliable;

Appropriate methods were used. The section needs to be improved as detailed below.

In the methodology section, line 107-108 the statement “Samples of spaced about 50m apart were randomly selected…” does not make grammatical sense. What did the authors mean?

Response: We eadjust the sentence content to–Samples were chosen with a spatial separation of more than 50 meters to avoid close kinship, and leaf materials were preserved by drying in silica gel.

Additional aspects of genetic diversity should be explored. Some important analysis such as linkage disequilibrium (LD) patterns and samples’ co-ancestry have not been done. The authors are encouraged to explore this option since it would provide information on the historical recombinations that could have occurred within samples as well as help in understanding the observed population structure.

Response: We added the PopLDdecay analyses in our study;

The grammar of this manuscript has been carefully checked and revised.
