# Supplementary material for: Population genetic diversity and structure of the endangered species Tetracentron sinense Oliver (Tetracentraceae) with SNPs based on RAD sequencing
Source: PLoS One. 2025 May 20;20(5):e0324161. doi: 10.1371/journal.pone.0324161 (PMC12091802; doi:10.1371/journal.pone.0324161)
Supplement: Attachment — Submitted filename: Response to Reviewer Comments.docx [file pone.0324161.s016.docx]

**Response to Reviewer Comments**

**Manuscript Title: Population genetic diversity and structure of the endangered species *Tetracentron sinense* Oliver (Tetracentraceae) with SNPs based on RAD sequencing**

**Dear Editor and Reviewer,**

I sincerely apologize for the delayed response to your comments. Due to recent health issues, I was hospitalized for an extended period, which prevented me from addressing your feedback in a timely manner. Below are the point-by-point responses to the issues raised:

**1. Methodology: Line 128–Explicit Declaration of De Novo Assembly**

**Reviewer Comment:**

‘The authors should declare explicitly that they did a de novo assembly for the benefit of readers unfamiliar with Stacks software.’

**Response:**

We have added an explicit statement in the Methodology section to clarify the de novo assembly process. Specifically, we revised Line 128 to:

"To ensure transparency, we explicitly declare that we performed de novo assembly of the genome using Stacks software (Catchen et al., 2013), a critical step for readers unfamiliar with the workflow."

This addition directly addresses the reviewer’s concern and enhances clarity for non-specialist readers.

**2. Spatial Population Genetic Analysis Using TESS**

**Reviewer Comment:**

‘Combine GPS coordinates with SNP data to explore ancestry estimation using TESS and add maps to the results.’

**Response:**

We initially planned to use TESS software for spatial population genetic analysis as recommended. However, due to open-source licensing restrictions encountered during implementation, we instead employed Adegenet v2.1.11 (Jombart, 2008) for principal component analysis (PCA) and population structure inference (Figure 2).

This analysis strengthens our conclusions about population origins and enriches the manuscript’s spatial genetic component.

**3. Replacement of Table 6 with an LD Scatter Plot**

**Reviewer Comment:**

‘Replace Table 6 with an LD scatter plot to visualize decay rate.’

**Response:**

We agree with the reviewer’s suggestion. Table 7 has been replaced with an LD scatter plot (Figure 4), accompanied by a detailed explanation in the Results section (Lines 318–322). The figure now clearly illustrates LD decay over physical distance, aligning with the reviewer’s recommendation.

**4. Language and Grammar Improvements**

**Reviewer Comment:**

‘Final draft requires proofreading by an English-speaking colleague.’

**Response:**

We have engaged a professional English-language editor (certificate attached) to review the manuscript for grammar, clarity, and style. Key improvements include:

- Revised sentence structures for flow and readability.

- Standardized terminology across sections.

- Removed redundant phrasing and ensured consistency in scientific reporting.

All language-related changes are tracked in the revised manuscript.

**Conclusion**

We believe these revisions address all reviewer concerns thoroughly. The manuscript now includes explicit methodological details, enhanced spatial analysis, improved visualizations, and polished language. We appreciate the reviewer’s constructive feedback and look forward to the next steps in the publication process.

Sincerely,

Zhongqiong Tian

bhgan@cwnu.edu.cn

lhagchong@utibet.edu.cn
